# Development and validation of a nomogram model for sleep disorders in patients with recurrent implantation failure based on physiological and lifestyle factors

**DOI:** 10.3389/fendo.2025.1585144

**Published:** 2025-08-21

**Authors:** You Zhang, Ningxin Qin, Jing Hu, Jie Bai, Mengjia Pan, Yan Xu, Xin Huang, Ke Wang

**Affiliations:** ^1^ Information Center, Shanghai Key Laboratory of Maternal Fetal Medicine, Shanghai Institute of Maternal-Fetal Medicine and Gynecologic Oncology, Shanghai First Maternity and Infant Hospital, School of Medicine, Tongji University, Shanghai, China; ^2^ Center of Reproductive Medicine, Shanghai Key Laboratory of Maternal Fetal Medicine, Shanghai Institute of Maternal-Fetal Medicine and Gynecologic Oncology, Shanghai First Maternity and Infant Hospital, School of Medicine, Tongji University, Shanghai, China; ^3^ School of International Medical Technology, Shanghai Sanda University, Shanghai, China; ^4^ Center of Reproductive Medicine, Xinhua Hospital Affiliated Shanghai Jiao Tong University, School of Medicine, Shanghai, China

**Keywords:** recurrent implantation failure of embryos, sleep quality, prediction model, LASSO regression, nomogram

## Abstract

**Objective:**

To establish and validate a nomogram model for the quality of sleep in patients with recurrent implantation failure (RIF) and to evaluate its performance.

**Methods:**

From January 2023 to June 2023, 484 RIF patients who underwent ART fertilization treatment at the Reproductive Medicine Center of Tongji University-affiliated Obstetrics and Gynecology Hospital were selected as the modeling set and internal validation. Additionally, from July to September 2023, 223 RIF patients who underwent ART fertilization treatment at the Reproductive Medicine Center of Tongji University-affiliated Obstetrics and Gynecology Hospital were chosen as the external validation set. Their clinical data was collected. Lasso regression was used to screen potential predictive variables and multifactor logistic regression analysis was conducted to determine the final predictors. A nomogram model was established, and the model was evaluated using methods such as plotting receiver operating characteristic (ROC) curves, calibration curves, Hosmer-Lemeshow goodness of fit test, and decision curve analysis.

**Results:**

Through Lasso regression and multifactor logistic regression, 7 predictors were identified, including FSH, E2, depression mood (moderate, severe), daily exercise time, sun exposure, caffeine intake, and shift work (>16h/w) for constructing the nomogram model. The AUC for the modeling set was 0.971 (95%CI:0.952∼0.989), for the internal validation set was 0.960 (95%CI:0.937∼0.979), and for the external validation set was 0.850 (95%CI:0.739∼0.960), indicating good predictive performance of the model.

**Conclusion:**

This study established and validated a nomogram model composed of 7 clinical indicators for sleep disorders in RIF patients. The predictors include both physiological indicators and daily lifestyle habits, demonstrating significant predictive value and clinical application efficiency. It can be used for early identification of potential sleep disorders in RIF patients, providing certain reference significance for clinical work.

## 1 Introduction

Recurrent Implantation Failure (RIF) is a complex clinical syndrome in patients undergoing Assisted Reproductive Technology (ART) treatments. According to the diagnostic criteria set by the Chinese Medical Association’s Committee on Reproductive Medicine, RIF is defined as: women under 40 who, after transferring at least 3 high-quality embryos in 3 fresh or frozen cycles, still have not achieved clinical pregnancy (1). This condition affects approximately 10% of *in vitro* fertilization-embryo transfer patients worldwide (2). RIF brings financial and psychological burdens to infertile couples. Its clinical management faces serious challenges. After repeated assisted pregnancy failures, such patients often experience compound psychological issues such as anxiety, depression, and stigma associated with infertility. These psychological stress responses form a vicious cycle with sleep disorders, not only reducing their quality of life but also further affecting the outcomes of assisted reproduction. Sleep disorders, as a multi-dimensional health issue, are characterized by persistent abnormalities in sleep quality and quantity, leading to significantly impaired daytime functioning (3). Studies have confirmed that abnormal fluctuations in sleep parameters during the ART treatment cycle are significantly negatively correlated with embryo implantation success rates (4). This discovery aligns with the emphasis on sleep health management in the “Healthy China Action (2019-2030)” (5), highlighting the necessity of sleep intervention in the field of reproductive medicine. However, a systematic assessment system for sleep health issues in the RIF population has not yet been established, and clinical attention to their sleep health management is urgently needed. Against this background, this study aims to construct a prediction model for sleep disorders in RIF patients, focusing on deciphering bio-psycho-social multi-dimensional predictors affecting sleep quality and establishing a clinically practical risk assessment system. This will provide a theoretical basis for early identification of high-risk factors for sleep disorders in such patients and the development of targeted intervention strategies.

## 2 Materials and methods

### 2.1 Selection of research objects

The research objects are RIF patients who underwent IVF/ICSI-ET/FET assisted reproduction at the Reproductive Medicine Center of Tongji University’s Affiliated Obstetrics and Gynecology Hospital from January 2023 to June 2023. Inclusion criteria: ①Meet the diagnostic criteria for recurrent implantation failure in “Chinese Expert Consensus on the Clinical Diagnosis and Treatment of Recurrent Implantation Failure” (1); ②The female reproductive system and physical examination are normal, without any other secondary diseases; ③Willing to participate in this survey research voluntarily. Exclusion criteria: ①Exclude patients with immune system diseases; ②Exclude patients with a history of mental illness, alcohol and other psychoactive substances (except tobacco, coffee, tea), combined chronic diseases (hypertension, heart disease, uncontrolled or poorly controlled diabetes, autoimmune diseases, etc.), and other serious systemic diseases; ③Exclude patients with chromosomal karyotype or gene abnormalities in either one or both spouses; ④Exclude patients with reproductive system diseases affecting fertility, such as intrauterine adhesions, endometritis, and uterine cavity effusion; ⑤Patients with mental illnesses who are currently taking medications that affect mood or sleep. The sample size was estimated according to the cross-sectional study sample size calculation formula (6).


$$\text{n}=\frac{Z^{2}\cdot P\cdot \left(1-P\right)}{\delta^{2}}$$

α=0.05, Z(1-α)/2 = 1.96; Previous surveys showed (6) P=0.46, controlling the sampling error δ to 10% of the total rate, δ=0.05 is, calculated that the sample size is at least 382 cases, considering the 10% invalid response rate, the total sample size required is 421 cases. To reduce potential confounding, we applied strict exclusion criteria. This study strictly controls the indications and complies with various laws and ethical principles, and has been approved by the Ethics Committee of the Obstetrics and Gynecology Hospital Affiliated to Tongji University (Ethical Number: KS21296).

### 2.2 Methods

#### 2.2.1 Research tools

① General information questionnaire, referring to the “Chinese Insomnia Diagnosis and Treatment Guide” (7) self-designed, including 17 items such as age, body mass index (BMI), education level, per capita monthly income, infertility duration, smoking history (>20 cigarettes/d), smoking duration, drinking history (40°∼68°>50 mL/d), drinking duration, caffeine-containing beverages (>450ml/d), whether there is a habit of sunbathing (outdoor activities>30min/d), average daily exercise duration, weekly shift work hours, daily dinner time, bedtime, nap time, and whether the sleep conditions are frequently changed. ② Self-rating Anxiety Scale (SAS) and Self-rating Depression Scale (SDS) (8): according to the symptoms of the patients in the past 1 week, the scores of the 20 items in the scale are summed to get the total raw score, and the standard score = raw score × 1.25; according to the results of Chinese norm, the standard score of SAS is 50 points, among which 50∼59 points are mild anxiety, 60∼69 points are moderate anxiety, and >70 points and above are severe anxiety; the standard score of SDS is 53 points, 53∼62 points are mild depression, 63∼72 points are moderate depression, and >72 points are severe depression. ③ The Chinese version of perceived stress scale (CPSS) (8). This scale was developed by Cohen et al. in 1983 and revised by Chinese scholars Yang Tingzhong et al. in 2003, with a Cronbach’s alpha of 0.780 and high structural validity. The scale has 2 dimensions and 14 items, with a total score of 11∼26 points representing a lower level of perceived stress, 27∼41 points indicating moderate stress, and >42 points indicating a high level. ④ Pittsburgh Sleep Quality Index (PSQI) (9). This scale evaluates the subjective quality of patients’ sleep in the past month from 7 dimensions (subjective sleep quality, bedtime, sleep duration, sleep efficiency, sleep disorders, use of sleep drugs, and daytime dysfunction), each dimension 0∼3 points, total score 0∼21 points, the higher the score, the worse the sleep quality. With a total score of 8 points as the boundary, <8 points is considered normal sleep quality, and ≥8 points is sleep disorder. ⑤ Serum sex hormone levels, 5mL of peripheral venous blood was collected from the researchers on the day of endometrial transformation, and the levels of follicle-stimulating hormone (FSH), luteinizing hormone (LH), prolactin (PRL), estradiol (E2), progesterone (P), and anti-Müllerian tube hormone (AMH) were detected by electrochemiluminescence.

#### 2.2.2 Data collection methods

Before the start of the survey, experts from the Department of Reproductive Medicine and psychotherapists will train the medical staff participating in the survey on relevant knowledge. With the consent of the research subjects, the researchers will distribute questionnaires and ask the patients to fill them out independently on the day of transplantation according to the actual situation, providing sufficient time and an independent environment, and collecting the questionnaires on the spot after checking that there are no missing items. Relevant laboratory data will be obtained through electronic medical records. To ensure the accuracy and completeness of the data, all the data obtained will be entered into Excel by two people and organized and analyzed. All participants signed written informed consent forms, and the questionnaire data and laboratory results were linked through anonymous IDs to ensure privacy protection.

#### 2.2.3 Statistical methods

Based on the distribution of the data, normally distributed measurement data are expressed as mean ± standard deviation ( $\bar{x}$ ± s); non-normally distributed data are expressed as median (interquartile range). Independent sample t-tests are used for comparisons between groups. Categorical data are presented as counts (percentages), and group comparisons use the χ2 test. Without replacement, random sampling is employed to select 80% of the cases as the modeling group, with the remaining 20% as the validation group. The R 4.2.1 software is utilized for LASSO regression analysis to screen for potential risk variables, and the final predictors are determined based on multifactor logistic regression analysis, constructing a nomogram model. To prevent overfitting, 10-fold cross-validation was performed during the LASSO regression process. Receiver operating characteristic (ROC) curves are drawn based on the validation group data to evaluate the model’s discriminability. Bootstrap resampling is conducted 500 times to draw calibration curves for internal verification of the model. The Hosmer-Lemeshow goodness of fit test assesses model accuracy, and decision curve analysis (DCA) evaluates the clinical effectiveness of the nomogram model. P<0.05 is considered statistically significant.

## 3 Results

### 3.1 Comparison of clinical data of RIF patients

The present study investigated a total of 497 RIF patients who met the inclusion and exclusion criteria. After cleaning the data and excluding outliers and patients with missing clinical information, a total of 484 valid samples were obtained. Among them, 233 RIF patients had sleep disorders (PSQI≥8 points), accounting for 48.14% of the total number of participants. Comparing the clinical data of the two groups, there were statistically significant differences (P<0.05) in 15 factors between the sleep disorder group and the normal sleep group, including years of infertility and whether they drank tea, as detailed in **Table 1**.

**Table 1:** Comparison of clinical data between the two groups of patients.

Item	Sleep normally (n=251)	Sleep disorders (n=233)	t/x2/Z	P
Age (year)	33.05 ± 4.09	33.37 ± 4.166	-0.845	0.398
BMI (kg/m^2^)	22.94 ± 3.56	23.01 ± 3.61	-0.196	0.844
Infertility duration	3 (2,5)	4 (2,5.5)	-4.394	<0.001
Educational level			1.405	0.495
High school and below	62 (24.70%)	68 (29.18%)		
College/Undergraduate	131 (52.19%)	111 (47.64%)		
Master’s degree or above	58 (23.11%)	54 (23.18%)		
Monthly income (Yuan)			2.156	0.541
0∼5000	65 (25.90%)	74 (31.76%)		
5001∼10000	82 (32.67%)	73 (31.33%)		
10000∼15000	56 (22.31%)	47 (20.17%)		
>15000	48 (19.12%)	39 (16.74%)		
Caffeine drinks			79.677	<0.001
NO	198 (78.88%)	91 (39.06%)		
YES	53 (21.12%)	142 (60.94%)		
History of Alcohol			6.121	0.013
NO	236 (94.02%)	204 (87.55%)		
YES	15 (5.98%)	29 (12.45%)		
Alcohol Age (year)			0.283	0.594
0~5	9 (60%)	19 (65.52%)		
>5	6 (40%)	10 (34.48%)		
History of Smoking			1.505	0.220
NO	246 (98.01%)	224 (96.14%)		
YES	5 (1.99%)	9 (3.86%)		
Smoking Age (year)			0.280	0.597
0~5	1 (20%)	3 (33.33%)		
>5	4 (80%)	6 (66.67%)		
Weekly shift work (h)			61.889	<0.001
None	198 (78.88%)	113 (48.50%)		
1∼16	28 (11.16%)	29 (12.45%)		
17∼32	14 (5.58%)	35 (15.02%)		
>32	11 (4.38%)	56 (24.03%)		
Daily dinner time			8.080	0.018
before 18:00	29 (11.55%)	39 (16.74%)		
18:00∼20:00	145 (57.77%)	105 (45.06%)		
after 20:00	77 (30.68%)	89 (38.20%)		
Daily sleeping time			38.478	<0.001
before 22:00	24 (9.56%)	47 (20.17%)		
22:00∼24:00	147 (58.57%)	72 (30.90%)		
after 24:00	80 (31.87%)	114 (48.93%)		
Daily nap time (h)			0.623	0.891
None	45 (17.93%)	47 (20.17%)		
<0.5	110 (43.82%)	103 (44.21%)		
0.5~1	70 (27.89%)	59 (25.32%)		
>1	26 (10.36%)	24 (10.30%)		
Frequently changing sleep conditions			9.623	0.002
NO	188 (74.90%)	144 (61.80%)		
YES	63 (25.10%)	89 (38.20%)		
Sunbathe			25.264	<0.001
NO	194 (77.29%)	218 (93.56%)		
YES	57 (22.71%)	15 (6.44%)		
Daily exercise time (h)			141.317	<0.001
None	33 (13.15%)	150 (64.38%)		
≤0.5h	93 (37.05%)	49 (21.03%)		
0.5∼1	84 (33.47%)	27 (11.59%)		
>1	41 (16.33%)	7 (3.00%)		
SAS			35.232	<0.001
None	126 (50.20%)	82 (35.19%)		
Mild	92 (36.65%)	69 (29.61%)		
Moderate	29 (11.55%)	60 (25.75%)		
Severe	4 (1.59%)	22 (9.44%)		
SDS			71.313	<0.001
None	136 (54.18%)	61 (26.18%)		
Mild	93 (37.05%)	82 (35.19%)		
Moderate	19 (7.57%)	64 (27.47%)		
Severe	3 (1.20%)	26 (11.16%)		
CPSS			16.506	<0.001
Low pressure	32 (12.75%)	20 (8.58%)		
Moderate pressure	165 (65.74%)	124 (53.22%)		
High pressure	54 (21.51%)	89 (38.20%)		
FSH (IU/L)	6.30 (5.02,7.67)	11.30 (9.15,13.90)	-16.181	<0.001
LH (IU/L)	3.97 (2.70,5.78)	4.02 (2.75,6.13)	-0.156	0.876
E2 (pg/ml)	96.87 (83.74,106.83)	75.27 (65.06,85.55)	-11.116	<0.001
PRL (ng/ml)	10.91 (8.17,15.26)	10.82 (8.38,15.75)	-0.589	0.556
P (ng/ml)	51.65 (44.27,58.27)	51.65 (42.86,60.67)	-1.100	0.271
T (ng/ml)	0.30 (0.20,0.52)	0.27 (0.19,0.46)	-1.283	0.200
AMH (ng/ml)	3.03 (2.39,4.28)	2.87 (2.18,3.57)	-3.858	<0.001

### 3.2 LASSO regression screening results

**Figure 1:**
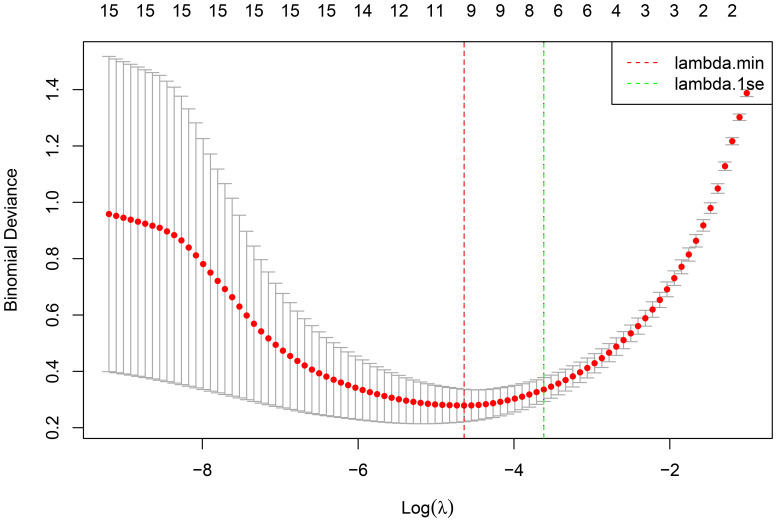
Screening of risk factors based on LASSO regression.

**Figure 2:**
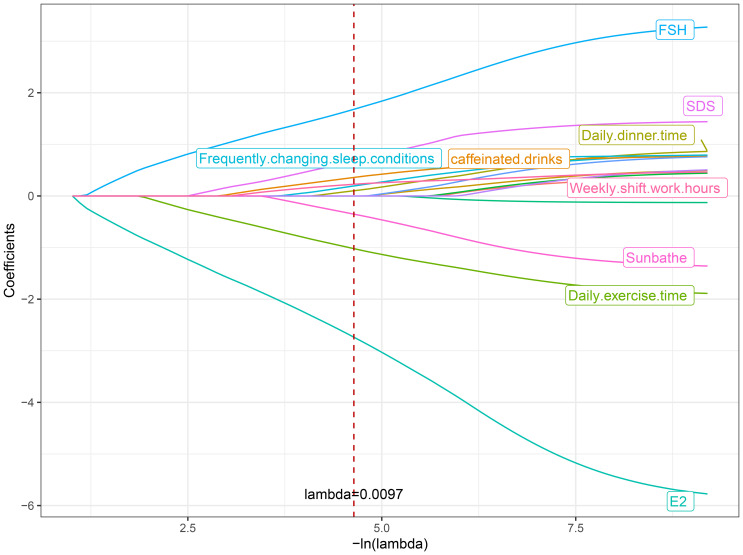
Coefficient distribution.

In the training set queue, LASSO regression was used to screen predictive variables. Through 10-fold cross-validation, based on the principle of minimizing mean squared error (MSE), we selected the λ with the smallest average error, namely lambda.min, as the optimal value (0.0097), thus ensuring optimal model complexity and generalization ability. Reduce the 15 influencing factors to 9 potential predictors, namely FSH, E2, Depression, Daily exercise time, Dinner time, Sun exposure, Frequent changes in sleep conditions, Caffeine drink intake, Weekly shift work hours, **Figures 1**, **2**.

### 3.3 Multifactor logistic regression analysis of sleep disorders in RIF patients

The application of multi-factor inclusion in Logistic regression was used to verify the above variables. Whether RIF patients developed sleep disorders was taken as the dependent variable, and the 9 factors screened by LASSO regression were used as independent variables for multi-factor Logistic regression analysis. The variable assignments are shown in **Table 2**. The results showed that FSH, E2, depressive mood (moderate, severe), daily exercise time, sun exposure, caffeine intake, and shift work (>16h/w) are 7 independent risk factors affecting sleep disorders in RIF patients (P<0.05). For details, see **Table 3**.

**Table 2:** Variable assignment for multi-factor logistic regression.

Variable	Assignment
Outcome variable	
Sleep disorders	0=NO, 1=YES
Predictor	
Depressive mood	0=None, 1= Mild, 2= Moderate, 3= Severe
Daily exercise time	0= None, 1 = 0-0.5h, 2 = 10.5~1h, 3= >1h
Daily dinner time	0=before 18:00, 1= from 18:00 to 20:00, 2= after 20:00
Sunbathe	0=NO, 1=YES
Frequently changing sleep conditions	0=NO, 1=YES
Caffeine drinks	0=NO, 1=YES
Weekly shift work	0=None, 1 = 0-16h, 2 = 17-32h, 3=>32h

**Table 3:** Multifactor logistic regression analysis of sleep disorders in RIF patients.

Independent variable	*B*	*SE*	*Wald χ^2^*	*P*	*OR*	*95%CI*
Constant	2.413	1.604	2.263	0.971	1.051	
FSH	0.636	0.094	46.250	<0.001	0.529	1.573~2.270
E2	-0.079	0.012	40.505	<0.001	1.083	0.901~0.947
Depressive mood (Using “None” as a reference)			20.215	<0.001		
Mild	0.379	0.417	0.824	0.364	1.461	0.645~3.310
Moderate	2.298	0.581	15.651	<0.001	9.950	3.188~31.060
Severe	2.558	0.884	8.361	0.004	12.905	2.280~73.053
Daily exercise time (Using “None” as a reference)			45.717	<0.001		
0~0.5h	-2.106	0.452	21.677	<0.001	0.122	0.050~0.295
0.5~1h	-3.029	0.511	35.071	<0.001	0.048	0.018~0.132
>1h	-3.520	0.756	21.702	<0.001	0.030	0.007~0.130
Daily dinner time (Using “before 18:00” as a reference)			2.726	0.256		
from 18:00 to 20:00	0.582	0.654	0.792	0.373	1.790	0.497~6.449
after 20:00	1.054	0.699	2.277	0.131	2.870	0.730~11.289
Sunbathe (Using “No” as a reference)	-2.246	0.608	13.667	<0.001	0.106	0.032~0.348
Frequently changing sleep conditions (Using “No” as a reference)	0.722	0.394	3.357	0.067	2.058	0.951~4.454
Caffeine drinks (Using “No” as a reference)	1.320	0.377	12.291	<0.001	3.745	1.790~7.835
Weekly shift work (Using “None” as a reference)			16.534	0.001		
0~16h	0.935	0.534	3.065	0.080	2.548	0.894~7.259
17~32h	1.396	0.662	4.452	0.035	4.039	1.104~14.772
>32h	2.476	0.683	13.141	<0.001	11.888	3.118~45.331

### 3.4 Construction of a nomogram model for sleep disorders in RIF patients

**Figure 3:**
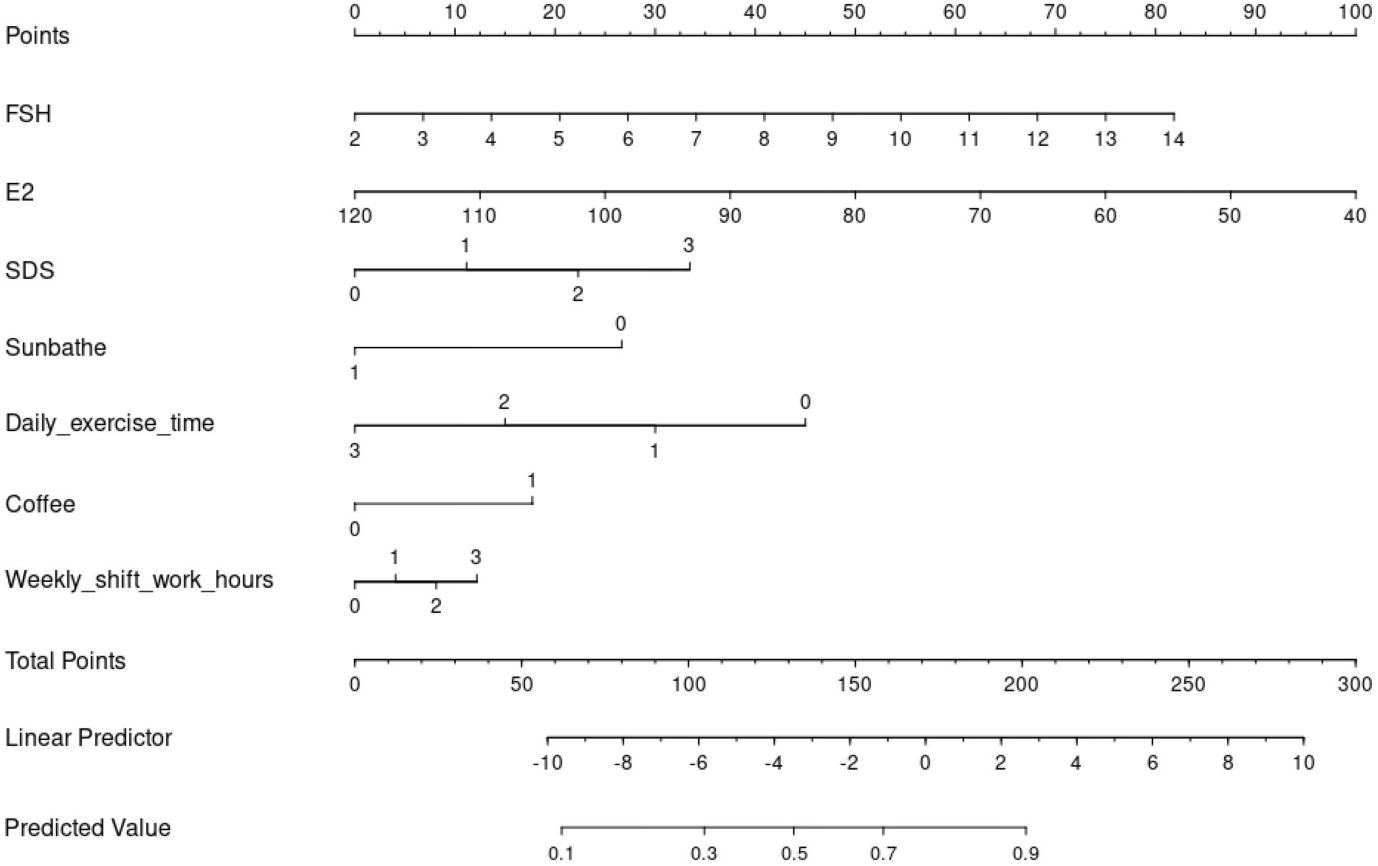
Nomogram model of sleep disorders occurring in RIF patients.

Using the R4.2.1 software, based on multi-factor Logistic regression analysis, the screened 7 predictive factors were included in the model to construct a nomogram model for sleep disorders in RIF patients. The total score ranges from 0 to 300 points, with the probability of occurrence of sleep disorders at the bottom line. The higher the total score, the higher the risk value. See **Figure 3**.

### 3.5 Evaluation of the nomogram model for sleep disorders in RIF patients

**Figure 4:**
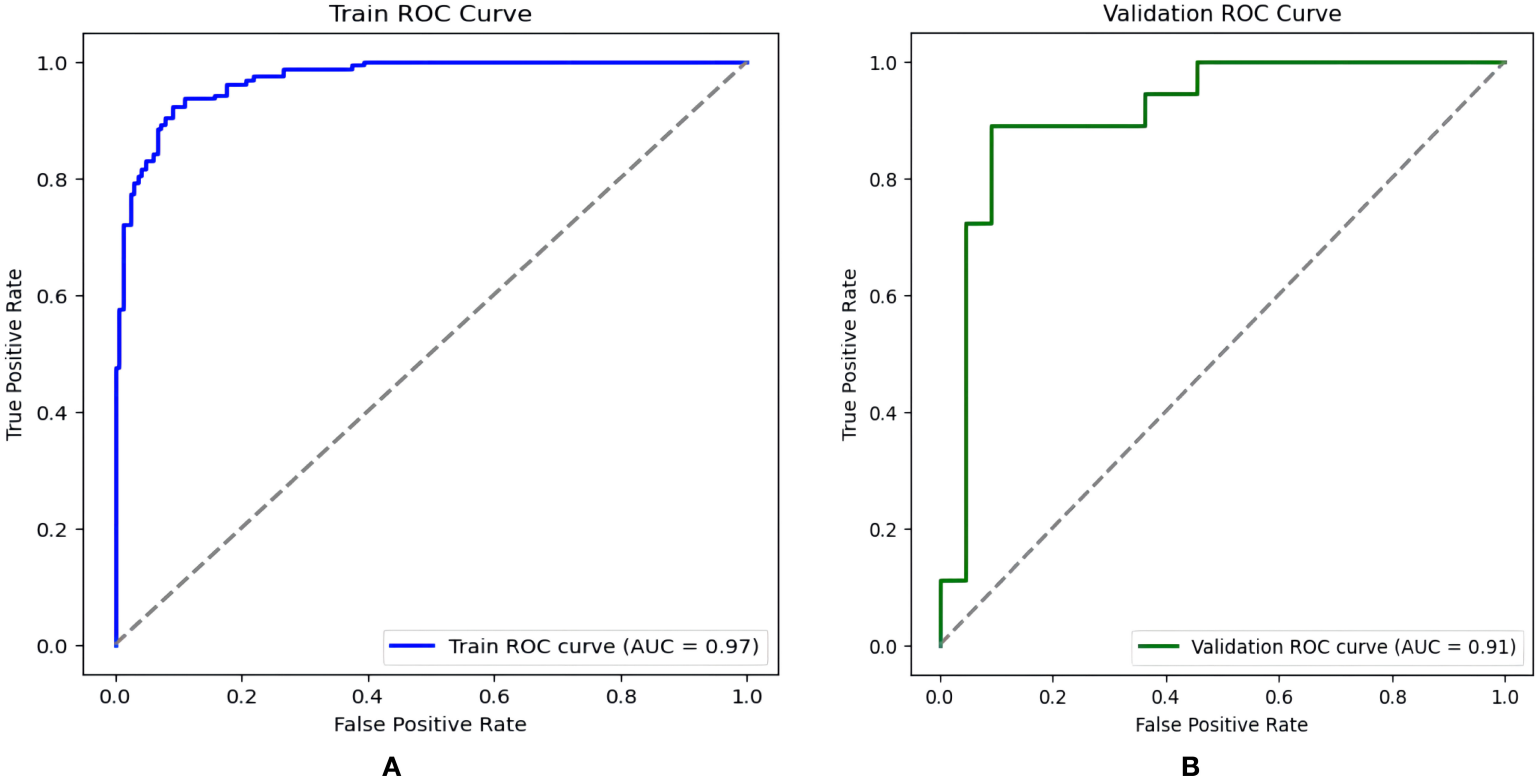
ROC curve of the sleep disorder nomogram prediction model in RIF patients. **(A)** training set **(B)** validation set.

**Figure 5:**
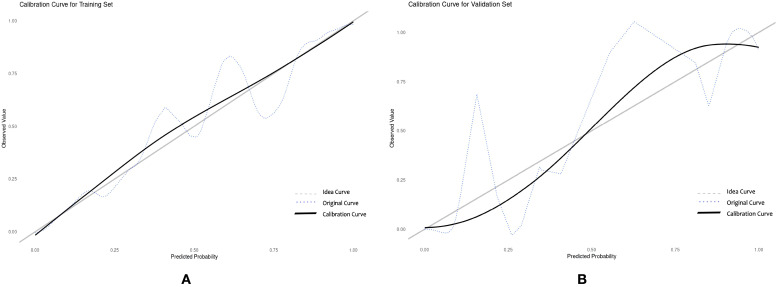
Calibration curve of the nomogram prediction model for sleep disorders in RIF patients. **(A)** training set **(B)** validation set.

**Figure 6:**
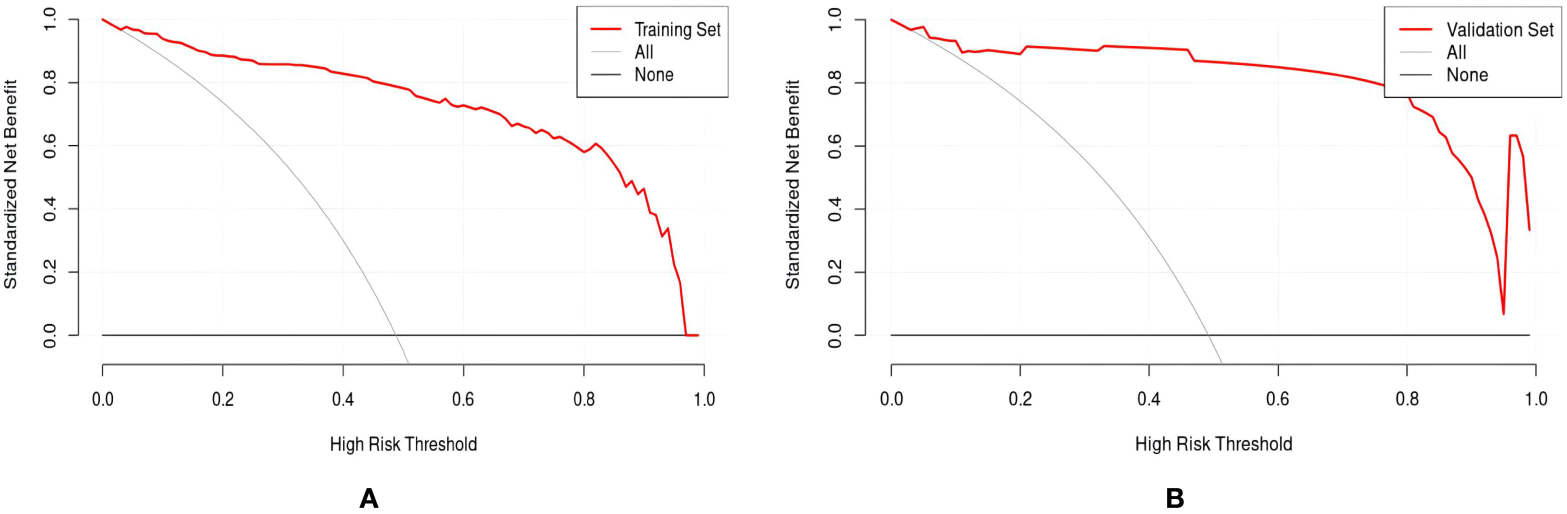
DCA curve of the sleep disturbance nomogram prediction model in RIF patients. **(A)** training set **(B)** validation set.

Drawing ROC curves to evaluate the discriminability of the model, the AUC of the modeling set is 0.971 (95%CI:0.952-0.989), with a sensitivity of 90.5% and a specificity of 90.9%. The AUC of the internal validation set is 0.912 (95%CI:0.824-0.969), with a sensitivity of 82.7% and a specificity of 90.9%, as shown in **Figure 4**. Drawing calibration curves of the model and conducting Hosmer-Lemeshow goodness-of-fit test: the P value of the Hosmer-Lemeshow test for the modeling set is 0.948, and that for the internal validation set is 0.903, indicating that the fit of this model is relatively good, as shown in **Figure 5**. Drawing DCA curves to assess the practical application of the model in clinical practice, the probability threshold for the modeling group is 0-97%, with a patient’s net return rate greater than 0, and the smaller the risk threshold, the greater the clinical net benefit; the probability threshold for the internal validation group is 0-95%, with a patient’s net return rate greater than 0, as shown in **Figure 6**.

### 3.6 External validation of the nomogram model for sleep disorders in RIF patients

**Figure 7:**
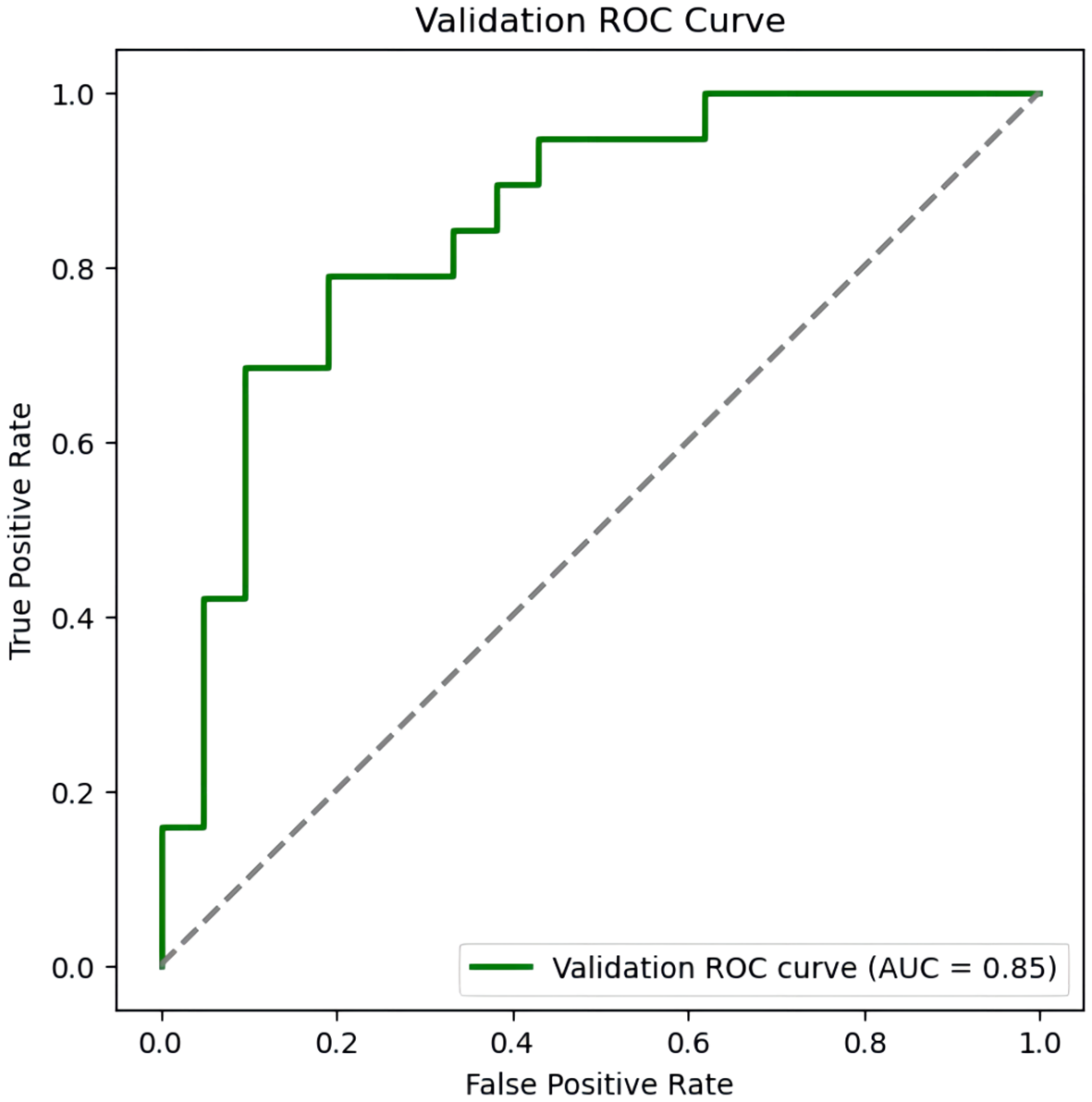
ROC curve of the external validation of the nomogram prediction model for sleep disorders in RIF patients.

From July to September 2023, 223 RIF patients who underwent assisted reproduction treatment and met the inclusion and exclusion criteria at the Reproductive Medicine Center of Tongji University-affiliated Obstetrics and Gynecology Hospital were selected. Among them, 88 RIF patients had sleep disorders (PSQI≥8 points), accounting for 39.46% of the total number of study participants. ROC curve analysis results showed that the AUC of the nomogram model predicting sleep disorders in externally validated RIF patients was 0.850 (95%CI:0.739∼0.960), with a sensitivity of 84.2% and a specificity of 66.7%, as shown in **Figure 7**. The Hosmer-Lemeshow test P=0.276.

## 4 Discussion

Patients with RIF may experience decreased sleep quality due to the stress of repeated assisted pregnancy failures and cyclical medication treatments, as well as the tendency to develop psychological issues such as anxiety and depression. On the other hand, sleep disorders can further affect the hormonal environment required for these patients to conceive, affecting the quality of oocytes and fertilized eggs, thereby affecting the clinical assisted pregnancy outcome. Foreign research has found that about 30% of women undergoing ART assisted pregnancy have poor sleep quality (10). In China, approximately 46% of women have poor sleep quality during embryo transfer (6). The results of this study show that 48.14% of RIF patients have sleep disorders during the embryo transfer period, which is slightly higher than foreign research and similar to domestic research, possibly related to a more focused inclusion of research subjects and differences in social customs and culture. The above data all indicate that sleep disorders are a common problem among RIF patients, and improving the sleep disorders of such patients and improving their quality of life has become one of the goals of reproductive medicine staff. At present, although there have been studies reported on the influencing factors of sleep disorders in infertile patients, the various influencing factors have not been integrated into the same model, making it difficult to achieve personalized prediction. Therefore, it is necessary to construct a special risk prediction model for sleep disorders in assisted pregnancy patients, to identify high-risk factors in advance, guide clinical medical staff to take early intervention measures, formulate personalized and scientifically effective intervention plans, thereby reducing the incidence of sleep disorders.

To ensure that the potential influencing factors of the model are more representative, this study uses LASSO regression to screen characteristic variables, avoiding multicollinearity (11). Based on LASSO regression, this study combined with multi-factor logistic regression to screen out 7 risk factors to construct a prediction model. The results show: the AUC of the modeling set is 0.971 (95%CI:0.952∼0.989), sensitivity is 90.5%, specificity is 90.9%, and Hosmer-Lemeshow test P=0.948; the internal validation set AUC is 0.912 (95%CI:0.824∼0.969), sensitivity is 82.7%, specificity is 90.9%, and Hosmer-Lemeshow test P=0.903; indicating that the model has good discriminability and internal consistency. In addition, the model performed equally well in external validation. In the external validation set, the AUC is 0.850 (95%CI:0.739∼0.960), sensitivity is 84.2%, specificity is 66.7%, and Hosmer-Lemeshow test P=0.276, suggesting that the model constructed in this study is scientific and has good predictive performance. At the same time, we employed a nomogram model to visualize related risk factors through line segments and integrated it into a scoring system to achieve individualized prediction for RIF patients with sleep disorders.

A total of 7 independent risk factors affecting the sleep quality of RIF patients were obtained in this study, namely FSH, E2, depression, daily exercise time, sun exposure, caffeine intake, and shift work. Fluctuations in reproductive hormones can affect sleep quality. Studies have pointed out that FSH is positively correlated with sleep time, while E2 is negatively correlated with sleep quality (12). E2 is involved in the regulation of a woman’s own body temperature and daily life rhythm. Low levels of E2 will affect normal temperature regulation, especially the cooling of the skin, leading to a decrease in deep body temperature, which can easily interrupt nighttime sleep and cause sleep disorders (13). Meanwhile, the level of FSH will increase with the decrease of E2. FSH may participate in sleep disorders through the feedback regulation process of E2. In addition, RIF patients undergo significant changes in hormone levels due to receiving estrogen medication during assisted reproductive therapy. Estrogen interacts with multiple neurotransmitters to affect the alternation of sleep wake cycles. Progesterone exerts a sedative effect on the central nervous system, while sex hormones regulate the hypothalamic pituitary gonadal axis, promoting compensatory increase in FSH secretion, increasing rapid eye movement sleep, reducing slow wave sleep, and disrupting sleep rhythms (14). Furthermore, the poor sleep quality of infertile patients may be related to psychological factors (15). In this study, mild depression did not show statistical significance for sleep disorders (P>0.05). Long-term negative emotions can cause disruptions in the cerebral cortex, leading to an increase in the excitability of the sympathetic nervous system and an increase in the concentration of catecholamines in the blood. This results in the body’s stress response to counteract negative emotions. When negative emotions exceed one’s psychological, it can easily lead to pathological states, resulting in sleep disorders (16). Anxiety and depressive moods can also lead to an imbalance in brain neurotransmitters. Changes in the levels of dopamine and norepinephrine may affect the stability of the sleep-wake cycle, leading to a decline in sleep quality (17). Additionally, lifestyle habits such as lack of sunlight exposure, lack of exercise, excessive intake of caffeine, and shift work (>16h/w) also affect a patient’s sleep quality. From a physiological perspective, bad lifestyle habits mainly affect women’s internal endocrine regulation, leading to poorer sleep quality in such patients. Light stimulation mainly regulates the activity of the nervous and endocrine systems and the secretion of hormones like melatonin to establish and consolidate regular sleep-wake cycles (18). Exercise promotes the further secretion of endorphins in the brain, bringing a sense of pleasure to the body, distracting patients from excessive focus on infertility issues, ensuring quality of life, actively coping with stress, and improving sleep quality (19). Changes in sleep patterns may disrupt the function of the hypothalamus-pituitary-gonadal axis, alter the secretion of gonadotropins and sex steroids, inhibit the production of melatonin, and interfere with reproductive processes. The disruption of circadian rhythms driven by hormonal imbalances (such as FSH/E2) and behavioral factors (such as shift work, caffeine) may impair the synthesis and secretion of melatonin, and melatonin deficiency is associated with poor sleep quality and reproductive dysfunction. This not only further affects sleep quality but can also adversely affect the outcome of ART fertility treatments (20).Caffeine can induce abnormal excitation of the nervous system by regulating the neurotransmitter system, leading to a certain degree of insomnia or anxiety-like states (21). At the same time, it can promote the secretion and release of excitatory neurotransmitter epinephrine from the adrenal glands into the bloodstream, jointly stimulating various physical tissues and the central nervous system, enhancing muscle contraction, promoting human excitation, and consequently leading to a decline in sleep quality (22).

However, this study still has certain limitations. This study utilizes the PSQI scale, a subjective tool, to assess sleep quality. Although this scale has been validated, due to practical operational constraints, objective sleep assessment methods such as polysomnography were not included. Additionally, the focus of this study is limited to single center RIF patients, which may lack universality and may result in errors in the predictive performance. Future research is encouraged to combine subjective and objective measurement methods to enhance accuracy, and conduct multicenter, large sample prospective studies to further validate the performance of predictive models.

## 5 Summary and suggestion

This study constructed and validated an early prediction model for sleep disorders in RIF patients through LASSO and multifactor logistic regression, consisting of 7 indicators. These include both physiological indicators and patients’ daily lifestyle, demonstrating significant predictive value and clinical application efficiency, which has certain reference significance for clinical work. The nomogram model can be integrated into clinical workflows through a digital calculator or app embedded within the electronic medical record system. Clinicians can input patient values to obtain real-time risk scores. Additionally, high-risk patients can be referred to sleep hygiene education programs, cognitive behavioral therapy for insomnia (CBT-I), or melatonin supplementation trials where appropriate.

## Data Availability

The data analyzed in this study is subject to the following licenses/restrictions: The dataset used in this study is not publicly available due to privacy and confidentiality restrictions. Requests to access these datasets should be directed to wangkeyfy@126.com.
